# Alternative polyadenylation of single cells delineates cell types and serves as a prognostic marker in early stage breast cancer

**DOI:** 10.1371/journal.pone.0217196

**Published:** 2019-05-17

**Authors:** Nayoung Kim, Woosung Chung, Hye Hyeon Eum, Hae-Ock Lee, Woong-Yang Park

**Affiliations:** 1 Samsung Genome Institute, Samsung Medical Center, Seoul, South Korea; 2 Department of Molecular Cell Biology, Sungkyunkwan University School of Medicine, Suwon, South Korea; 3 Department of Health Sciences and Technology, Samsung Advanced Institute for Health Sciences &Technology, Sungkyunkwan University, Seoul, South Korea; 4 GENINUS Inc., Seoul, South Korea; University of South Alabama Mitchell Cancer Institute, UNITED STATES

## Abstract

Alternative polyadenylation (APA) in 3’ untranslated regions (3’ UTR) plays an important role in regulating transcript abundance, localization, and interaction with microRNAs. Length-variation of 3’UTRs by APA contributes to efficient proliferation of cancer cells. In this study, we investigated APA in single cancer cells and tumor microenvironment cells to understand the physiological implication of APA in different cell types. We analyzed APA patterns and the expression level of genes from the 515 single-cell RNA sequencing (scRNA-seq) dataset from 11 breast cancer patients. Although the overall 3’UTR length of individual genes was distributed equally in tumor and non-tumor cells, we found a differential pattern of polyadenylation in gene sets between tumor and non-tumor cells. In addition, we found a differential pattern of APA across tumor types using scRNA-seq data from 3 glioblastoma patients and 1 renal cell carcinoma patients. In detail, 1,176 gene sets and 53 genes showed the distinct pattern of 3’UTR shortening and over-expression as signatures for five cell types including B lymphocytes, T lymphocytes, myeloid cells, stromal cells, and breast cancer cells. Functional categories of gene sets for cellular proliferation demonstrated concordant regulation of APA and gene expression specific to cell types. The expression of APA genes in breast cancer was significantly correlated with the clinical outcome of earlier stage breast cancer patients. We identified cell type-specific APA in single cells, which allows the identification of cell types based on 3’UTR length variation in combination with gene expression. Specifically, an immune-specific APA signature in breast cancer could be utilized as a prognostic marker of early stage breast cancer.

## Background

Alternative polyadenylation (APA) in 3’ untranslated regions (3’UTR) is a major post-transcriptional mechanism, controlling gene expression by influencing transcript abundance, cellular localization, and interaction with microRNAs [1]. Recent studies have found that the change in 3’UTR length is tightly associated with the regulation of cell proliferation as well as differentiation during an immune response [2] and cancer growth [3]. The usage of shorter 3’UTRs via APA events is most common (91%) in cancer and occurs on a global scale [4]. There has been an increase in the trial to systematically detect APA events across diverse cancers including breast cancer [3, 5]. Especially, APA usages in specific genes and transcriptional signalings, such as PRELID1 [6], USP9X, SNX3, and YME1L1D [7], have been reported as a strong predictor of clinical outcomes in the breast cancer. Although the biological significance is widely accepted, its clinical application as a prognostic biomarker or therapeutic target is not fully evaluated. Thus, understanding the regulation of expression via APA events across diverse cell types may provide new insights into cancer therapeutics.

Recently, a range of algorithms has been developed to quantify relative changes in 3’UTR length using RNA sequencing data to infer APA events. There are two classes of analytical methods created for the identification of de novo 3’UTR sites. Algorithms such as Dynamic analyses of Alternative PolyAdenylation from RNA-Seq (DaPars) [4] and 3USS [8] were developed to identify the locations of novel 3’UTR sites, whereas predefined APA sites from public databases were utilized in Roar [9], MISO [10], and ChangePoints [11], etc. The combined use of those methods can provide an opportunity to identify novel and reliable 3’UTR APA events using large-scale RNA sequencing datasets.

Single-cell RNA sequencing is primarily used to explore intratumoral heterogeneity in gene expression. Detecting cell-to-cell variations in polyadenylation site usage has been suggested [12], but single-cell level analysis for APA events has rarely been explored on a large scale. Single-cell RNA sequencing datasets in diverse cancer types can be found in public repositories like JingleBells [13] and scRNASeqDB (https://bioinfo.uth.edu/scrnaseqdb/). Especially, full-length single-cell RNA sequencing data contains genome-wide reads that permit prediction of relative 3’UTR usage. The APAs inferred from those predictions allow comprehensive profiling of alternative 3’UTRs at the single-cell level.

In the present study, we analyzed a single-cell RNA sequencing dataset containing 515 cells from 11 breast cancer patients to profile comprehensive APA events across diverse cell types. We characterized 1,176 functional categories and 53 genes associated with shorter 3’UTR usage and over-expression specific to each cell type. Most of gene sets (94.7%) were unique, and only partial gene sets (5.3%) were recurrently identified in multiple cell types. Among the gene sets, those associated with proliferation marked distinct APA events strictly confined to specific cell types. Furthermore, the expression level of those gene signatures was significantly associated with patient survival. Finally, the APA profiles on 598 tumor cells covering breast cancer, glioblastoma, and renal cell carcinoma distinguished tumor samples belonging to the cancer type. Therefore, the large-scale single-cell analysis demonstrates that cell type and cancer type-specific transcriptional regulation is associated with APA signals.

## Materials and methods

### Data acquisition

The raw RNA-seq data for 515 cells from 11 breast cancer patients [14] and 34 cells from a renal cancer patient [15] were obtained from the NCBI Gene Expression Omnibus database under the accession codes GSE75688 and GSE73122. Raw RNA-seq data for 355 cells from 3 glioblastoma patients were downloaded from European Genome-phenome Archive (EGA) with accession code EGAS00001001880 [16]. The data were generated by C1 Single-Cell Auto Prep System (100–5760, Fluidigm, San Francisco, CA, USA) as full-length transcripts. Detailed information of clinical parameters, cell types, and samples for the acquired datasets were described in S1 Table. To estimate the usage of 3’UTR length, we generated the .bam file as an input for the method Roar using the 2-pass mode of STAR_2.4.0b (default parameters) [17]. We generated .bedgraph file as an input for the method DaPars from the .bam file using the ‘genomCoverageBed’ command (BEDtools v2.17.0) [18]. For the expression analysis in breast cancer, we extracted 34,942 genes for which expression values were present in at least one cell. Relative expression of each gene is represented by transcripts per million (TPM) using RSEM v1.2.17 (default parameters) [19]. As a normal reference, we obtained the raw RNA-seq data of normal breast, brain, and kidney tissues from the Body Map 2.0 project from ArrayExpress (Query ID: E-MTAB-513, available from http://www.ebi.ac.uk/arrayexpress). Sequential methods for read alignments and quantification were applied in accordance with the process of preparing single-cell RNA-seq data. For survival analysis, RNA-seq and clinical data from patients’ Breast Invasive Carcinoma (BRCA) samples were obtained from The Cancer Genome Atlas (TCGA). This RNA-Seq data (Level 3) included 1,073 (updated on 2017) tumors, and the expression of each gene is represented as upper quartile normalized RSEM (RNA-Seq by Expectation Maximization) count estimate. And the RNA-seq BAM files for 110 BRCA/normal breast pairs were downloaded from the GDC Data Portal (https://portal.gdc.cancer.gov/). Subtypes of BRCA tumors were predicted using the R package ‘genefu.’

### Estimation of usage of 3’UTR length

APAs were estimated by length changes of 3’UTR using two complementary methods, DaPars (default parameters) [4] and Roar [9]. DaPars searches all regions of 3’UTR in the reference genome (hg19), while Roar uses the .gtf files generated from public APA databases of PolyA_DB2 [20] using the given single-cell and bulk RNA-seq samples.

### Transformation of gene-level data into gene sets

The APA estimates of ‘change in Percentage of Distal polyA site Usage Index’ (ΔPDUI, by DaPars) and ‘Ratio of A Ratio’ (roar, by Roar) were separately used as input data. Gene expression quantified as TPM was log2 transformed after plus 1. To assess the APA regulation and gene expression based on pathway activation, all gene symbols were matched with EntrezID and then applied to ssGSEA (using options in R packages called as ‘GSVA’) to calculate an enrichment score per gene set. A total of 5,917 Gene Ontology (GO) terms were collected for ssGSEA referring to a gene set database, MSigDB v6.0.

### Selection of variable components

For the selection of variable components (genes and gene sets) for hierarchical clustering and dimensional reduction, we scored each gene and gene set for the variation level, defined as standard deviation (s.d.) across all single cells. Variable components were then identified as those with the variation level above the baseline, the mean and s.d. of all components. For gene-level analysis, we additionally filtered out genes estimated in less than 30% of all single cells.

### Statistical analysis to select cell-type specific signatures

To compare gene expression and APA levels of various cell types, Pearson’s correlation coefficient (PCC) was calculated in the scale of enrichment score for gene sets. To determine the statistical significance of PCC, we calculated p-values based on Fisher's Z transformation. To describe the enriched association of 3’UTR shortening and over-expression with a cell type, we calculated an odds ratio (OR) by quantifying a given cell population. All single-cells were classified into 4 groups–(A) cells showing 3’UTR shortening and over-expression on a cell type, (B) cells showing 3’UTR shortening and over-expression on the others, (C) cells not showing 3’UTR shortening and over-expression on a cell type, (D) cells not showing 3’UTR shortening and over-expression in the others. The median APA level and gene expression were used to determine whether a cell represented 3’UTR shortening and over-expression for each gene set and gene. Then, the Fisher exact test was used to determine the statistical significance of the agreement between individual query pairs [21]. For the selection of hits, we applied the cutoffs for PCC > 0 (p-value < 0.05) and OR > 2 (p-value < 0.01).

### Selection of tumor-specific gene sets

To select gene sets showing significantly switched 3’UTR specific to each cancer type, delta and t-test p-value were calculated using APA prediction data transformed into the gene-set. The delta of a gene set is given by the difference between the average of single cells for a given tumor type and the others. The significance (p-value) was calculated by two-sided t-statistics.

### Network-based clustering of gene sets

Clusters based on biological functions of gene sets were graphically organized into an interaction network using Cytoscape (v3.5.1) [22]. The edges in the network were determined by Jaccard index as intersection over union for the number of shared parent GO terms between two gene sets. The distances between nodes (gene sets) were defined using a force-directed layout.

### Survival analysis

For the refined analysis of survival rate, we acknowledged patient survival if the time of death after diagnosis was longer than 10 years. The tumor samples were divided into two classes along 25th and 75th percentiles of expression for each target gene. Survival curves were fitted using a Kaplan–Meier formula in the R package ‘OIsurv.’ Additionally, we performed multivariate Cox regression to investigate the relative risks in the R package ‘survival.’ The regression model was constructed considering 10 events such as age, race, pathologic stage, tumor weight, the presence or absence of ER/PR/Her2 and an indication of drug/radiation regimen, and expression classes of each gene.

## Results

### Cellular heterogeneity of 3’UTR length changes

The availability of full-length single-cell RNA sequencing data from patient tumor tissues provides the opportunity to predict and compare the changes in 3’UTR length among diverse cell types. We applied two complementary methods to determine the shortening and lengthening of 3’UTRs. DaPars scans all regions of 3’UTR in genes to detect novel APA sites [4], while Roar focuses on the known APA sites of 3’UTR to improve the sensitivity [9]. We mainly utilized the results of 3’UTR switching estimated from DaPars.

**Fig 1:**
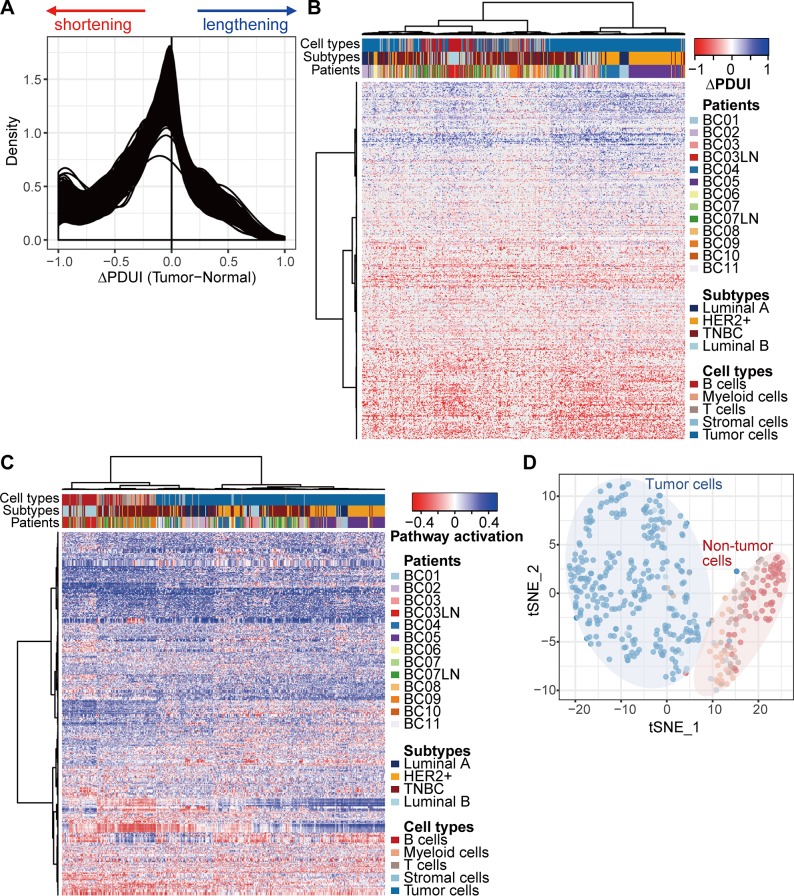
Different APA patterns between tumor and non-tumor cells. (A) The signals of APA (ΔPDUI calculated by DaPars) for all genes were shifted toward 3’UTR shortening in 515 single cells derived from 11 breast cancer patients (BC01-11). We followed the labels for the patients and cell types pre-defined to original datasets [14]. BC; Breast cancer, LN; Lymph nodes. Hierarchical clustering was performed using APA estimates (B) for genes and (C) gene sets on individual cells derived from breast cancer patients. Only 419 cells with at least 542 (10% of total genes) detected genes were selected for further analysis. A total of 1,452 genes and 3,353 gene sets showed the signals of APA on 419 cells. Among theme, 453 genes and 555 gene sets were selected as variable components for hierarchical clustering and dimensional reduction, respectively (see Methods). (D) Unsupervised tSNE on the gene set-level APAs separating 280 tumor and 139 non-tumor cells (immune and stromal cells) in breast cancer. Individual cells are colored for samples, consistent with the coloring in Fig 1B and 1C.

In this study, we used full-length single-cell RNA sequencing data generated in breast cancer patients’ tumor tissues to compare the APA regulations in diverse cell types [14]. Widespread shortening of 3’UTR is a global trend in cancer [3]. As expected, 3’UTR shortening was dominant in all cells derived from breast cancer patients’ tumor tissues (Fig 1A and 1B, S1A Fig). To increase the resolution of pattering, overall APA patterns were compared at the level of gene sets using single sample GSEA (ssGSEA). Clustering based on APA profiles in gene sets showed subgroups divided into tumor and non-tumor cells (Fig 1C and 1D, S1B Fig). No differences were associated with cancer subtypes or sample batches.

### Unique APA regulation among cell types

To identify the functional categories associated with diverse cell types, we utilized two metrics to determine the associations between 3’UTR usage and expression regulation in different cell types. Correlation coefficient reflects the global relationship of expression and 3’UTR length change in each cell type, while the odds ratio quantifies the specificity of 3’UTR shortening and expression of a gene set within each cell type (See methods for details). These two metrics allowed us to prioritize the cell type-specific gene sets for further study.

**Fig 2:**
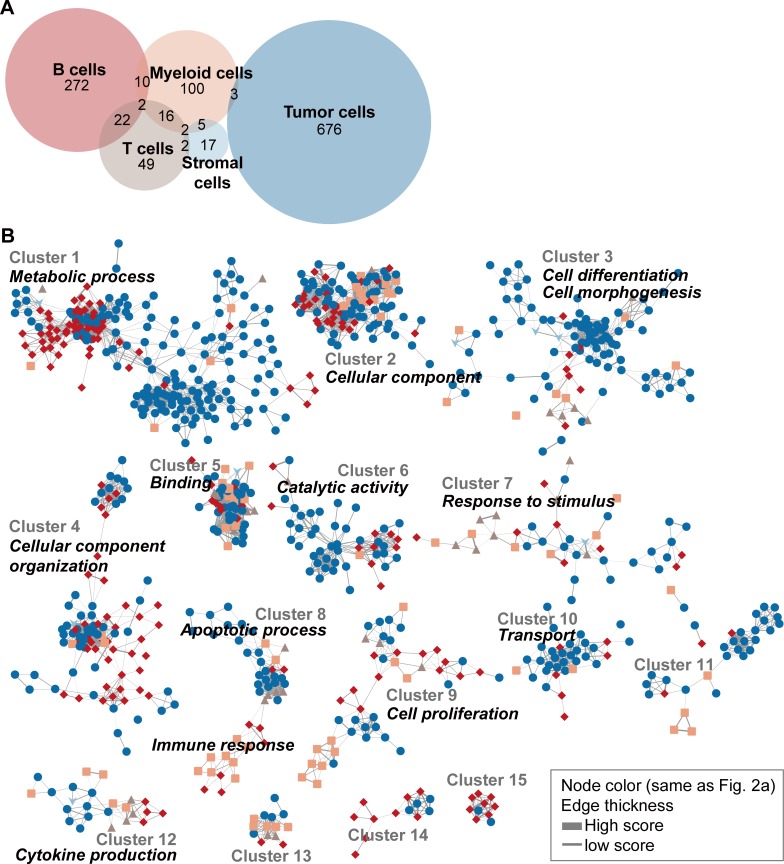
Cell type-specific functional categories associated with 3’UTR shortening and overexpression. (A) Venn diagram for 1,176 gene set hits specific to 5 cell types. Cell type specific gene sets were selected by correlation coefficient and odds ratio. (B) Network-based functional analysis of the selected gene sets. A total of 906 gene sets with sufficient interactions (> 0.5 Jaccard index and > 9 gene sets in a cluster) were selected for display. The cluster annotations of each gene set are available in S2 Table.

**Fig 3:**
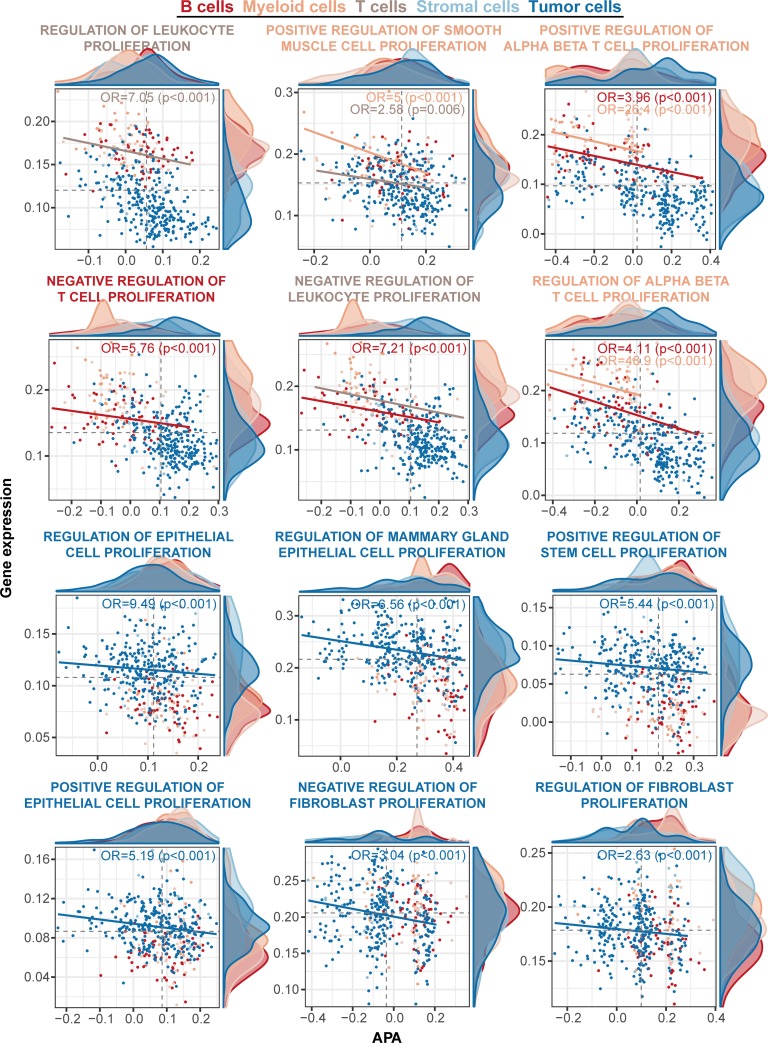
Cell type-dependent 3’ UTR shortening and expressional changes for genes associated with proliferation. The associations between 3’UTR switching and gene expression were compared for 12 gene sets classified into cellular proliferation. Dots in the plot represent 419 single-cells and are colored for each cell type. Odds ratio (OR) and significance are marked for specific cell types in the top right of the plot. The solid lines denote the fitted line constructed by generalized linear model (R function ‘glm’) and are colored for the matching cell types.

Consequently, we classified 1,176 gene sets specific to 5 main cell types of tumor, B lymphocytes, T lymphocytes, myeloid, and stromal cells (Fig 2A, S2 Table). Most of the hits were distinct among the cell types except for partial (5.3%) gene sets, which were recurrently selected in multiple cell types. These selected gene sets were clustered on the basis of their annotated GO terms in the network (Fig 2B, S2 Table). As a result, we observed dominant functional categories assigned to each cell type. Tumor cell-specific gene sets were widely distributed in diverse biological events such as apoptotic signaling, cell morphogenesis, and metabolic process. Previous work using deep sequencing of 3’UTRs of mRNA has shown that genes with switched APA sites are enriched in pathways including cell cycle, apoptosis, and metabolism in breast cancer cell lines [23]. Immune and stromal cell-specific gene sets were clustered together as functional categories of ‘immune response’ and ‘response to stimulus’. In particular, the clustering pattern of gene sets specific to 4 cell types except for stromal cell was exhibited in an event of cell proliferation. General shortening in 3’UTR length is tightly related to states of cellular proliferation and dedifferentiation [1]. We further confirmed that 3’UTR shortening and gene expression for gene sets classified into cell proliferation showed correlation patterns restricted to the associated cell type (Fig 3). This result suggests that APA linked to expression regulation is highly dependent on unique cellular lineages. Thus, the understanding of APA at single-cell resolution is useful to recognize the difference in transcriptional regulation among diverse cells in cancers.

### Distinct APA regulation among cancer types

**Fig 4:**
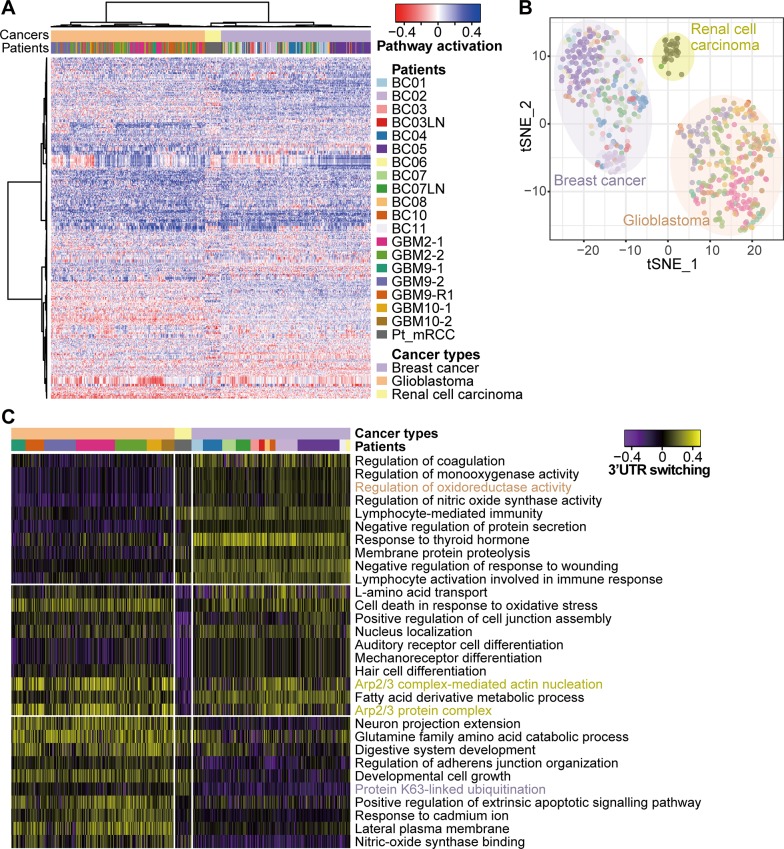
Heterogeneity of APA regulation among cancer types. (A) Hierarchical clustering using gene set-level APA estimates on 598 single-cells derived from 3 cancer types (280 tumor cells from 11 BC patients, 288 tumor cells from 3 GBM patients, and 30 tumor cells from 1 RCC patients). BC; Breast cancer, GBM; Glioblastoma, RCC; Renal cell carcinoma, T1,T2: Multiple regions in the brain, RLPS; Relapse. A total of 417 gene sets were used as variable components for hierarchical clustering and dimensional reduction (see Methods). (B) Unsupervised tSNE on the gene set-level APAs separating tumor cells along with cancer types. The dot colors represent patients which are the same as in Fig 4A. (C) APA map of top10 gene sets significantly (p<0.01) switched in each cancer type. The colors for cancer types and patients are the same as in Fig 4A.

Previous work has reported that regulation of APAs varies between tissues [24]. To assess APA variations among tissues and individual patients, we collected more public full-length single-cell RNA sequencing data generated from glioblastoma [16] and renal cell carcinoma [15] tissues. For the comparison of tumor cell types, we only used the APA-predicted data in 280 tumor cells from breast cancer patient’ tumor tissues. Clustering based on 3’UTR length changes at the level of gene sets clearly distinguished tumor tissues (Fig 4A and 4B). We further investigated the gene sets contributing to tumor-specific clusters through differential APA regulation. We identified 739, 898, and 731 gene sets significantly (p<0.01) switched in breast cancer, glioblastoma, and renal cell carcinoma, respectively (S3 Table). Sorting through the delta, we compiled a list of the most differential gene sets (top10 hits) in each cancer and confirmed discriminative enriched patterns toward 3’UTR shortening (Fig 4C). The biologic functions highlighting the listed gene sets are known to play important roles in carcinogenesis for each cancer. For example, oxidoreductase family genes were reported as therapeutic targets in brain tumor [25, 26]. A k63-linked ubiquitination is the major modulation inducing specific expression of calmodulin-like protein 5 in patients with primary breast cancer [27]. In addition, the role of the Arp2/3 complex was experimentally validated in the movements of kidney cells [28]. These results suggest that the features of APA events are distinctively classified at the level of tissues, but not individuals.

### Clinical relevance of APA-associated marker genes

**Fig 5:**
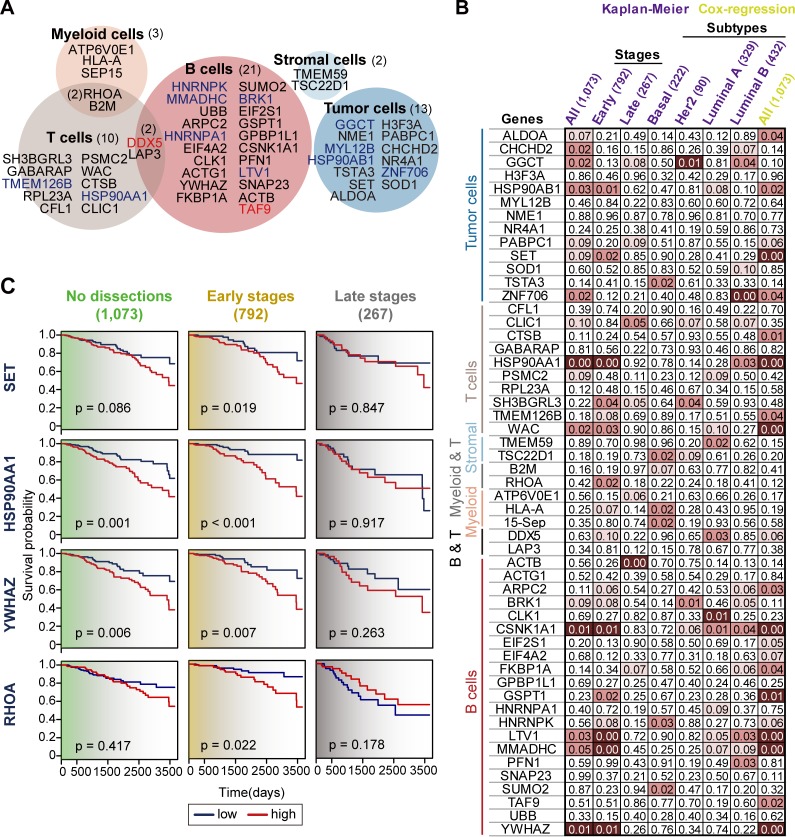
Cell type specific genes with clinical associations. (A) Venn diagram for 53 gene hits specific to 5 cell types. Red-colored genes were concurrently identified in the results of Roar. Blue-colored genes are de novo markers not predefined in a public APA database. Number in the bracket indicates the number of gene hits in each cell type category. (B) Kaplan-Meier and Cox regression analyses for 53 genes in TCGA BRCA patients. Variables showing a significant difference in survival rate are highlighted in symbols (light red; p<0.01, red; p<0.05, and dark red; p<0.1). Number in the bracket indicates the number of tissue samples in each subset. (C) Kaplan-Meier survival plot for *SET*, *HSP90AA1*, *YWHAZ*, and *RHOA* in TCGA BRCA patients. The tumor samples were annotated as ‘high’ and ‘low’ (25th and 75th percentiles, respectively) groups for the expression signal of each gene. The p-value was determined by the Log-Rank test. The tumor tissue samples were categorized into early and late cancer stages (Stage I and II / III and IV) based on the ‘pathologic_stage’ information in the clinical dataset. Number in the bracket indicates the number of tissue samples in each subset.

To clarify the associations between gene markers and APA regulation, we re-analyzed single-cell sequencing data from breast cancer patients at the gene level. We found 53 genes associated with shorter 3’UTR usage and over-expression specific to each cell type (Fig 5A, S4 Table). Consistent with the gene set-level results, most of the hit genes were distinct among cell types except for 4 overlapping genes for immune cell types. To explore the clinical impact of those genes, we performed Kaplan-Meier survival analysis and examined the association of the expression changes and survival in breast cancer patients using TCGA RNA sequencing data. The tumor samples were annotated as ‘high’ and ‘low’ (25th and 75th percentiles, respectively) along with the expression signal of each gene. In this analysis using all 1,073 BRCA samples, 10 of 53 genes showed significantly (p<0.05) different survival rates between the two groups (Fig 5B and 5C). The examples of read density for four selected genes (*SET*, *HSP90AA1*, *YWHAZ*, and *RHOA)* is clearly lower in each single-cell on the portion after proximal APA site (S2 Fig). By changing sample windows along the cancer stages, we confirmed that expression levels for a total of 11 genes significantly (p<0.05) affected survival rate in early stage tumors. In stark contrast, only 2 genes affected survival under the cutoff p-value of 0.05 in late-stage tumors. There was no difference in survival for molecular subtypes of breast cancer. Additionally, to overcome the effects of confounding factors such as cancer stage, age, and race, we performed multivariate Cox regression analysis, which estimates survival probabilities using more than one variable for modeling. Among 11 genes denoted as significant from Kaplan-Meier survival analysis in tissue samples, we demonstrated that the expression levels for 10 genes were independent (p<0.05) factors affecting survival in BRCA patients.

Our data mining results suggest that cell type-specific genes showing 3’UTR shortening and over-expression harbor clinical relevance. The functions of the selected genes are tightly connected with the specific cell type (Fig 5C). For example, over-expression of *SET* (SET nuclear proto-oncogene), defined as a tumor cell-specific signature, has been demonstrated in 50–60% of breast cancer cases [29]. The study suggested inhibition of SET as a potential antitumor strategy in breast cancer [29, 30]. Heat Shock Protein 90 (*HSP90*) is a T lymphocyte-specific signature and is known as a regulator of LAT (Linker of Activated T cells), which induces T cell activation [31–33]. A recent study provided strong evidence that the combination of immunotherapy and HSP90 inhibitors augments T-cell mediated anti-tumor response [34]. B cell-derived 14.3.3 protein zeta/delta (*YWHAZ*), defined as a B-lymphocyte specific signature, plays an important role in T cell trafficking [35]. Finally, Ras homolog gene family, member A (*RHOA*) is a T and Myeloid cell specific signature and is pivotal for the functioning of T cells[36] and macrophages[37]. Taken together, these results show that APA-associated gene signatures have a biological and clinical impact and warrant further investigations as prognostic and therapeutic targets.

## Discussion

Single-cell RNA sequencing provides an opportunity to investigate cell type specific connections among different genomic features. For instance, prior scRNA-seq studies of tumors have leveraged genetic features and polymorphism estimated from full-length transcriptomic data [38, 39]. Here we present a computational approach to explore transcriptional signatures associated with alternative polyadenylations in 3’UTR in combination with gene expression regulations at single cell resolution. The single cell approach solves the complexity issues coming from bulk RNA sequencing which contains tumor, stromal, and immune components of tumor tissues. We exploited only high-depth and full-length scRNAseq, as massively parallel 3’ or 5’ scRNAseq data have sparse read coverage and densities which prevents an accurate polyadenylation analysis.

We recognize that even full-length scRNA-seq data has a limitation in assessing the full spectrum of 3’UTR switching for all genes within a cell. When we applied gene set level analysis, we could find transcriptional trends associated with APAs which largely separated tumor and non-tumor cells. For this, we combined complementary metrics reflecting cell-type labels inferred from gene expression data, gene expression levels, and change of 3’UTR lengths. The enrichment trends demonstrated discriminative biologic functions specific to cell types of tumor vs. their neighboring immune and stromal cells. Especially, signals of APA and gene expression for functional categories of ‘the regulation of cell proliferation’ were strikingly different among cell types (Fig 3), which confirmed the connection between APA and cellular proliferation in cancer and the immune system [2, 40].

Previous work suggested that a 3’UTR-based classifier could improve prognostic performance in triple-negative breast cancer patients [41]. To search for clinically relevant signatures in APA, we first selected 53 genes showing 3’UTR shortening and over-expression specific to each cell type (Fig 5A). Among these cell type-specific signatures, Kaplan-Meier and Cox regression analyses confirmed outstanding risk stratification by 11 genes for patients with BRCA tumors (Fig 5B and 5C). Although their 3’UTR shortening is clearly represented at the single-cell level (S2 Fig), bulk RNA-sequencing in TCGA BRCA failed to demonstrate the association of the change of APA signals and expression level of those genes (S3 Fig). As bulk RNA-sequencing represents the average of all cell categories in tumor tissues, the difference in read density between tumor and normal samples might have been mixed and burred. In addition, we cannot rule out the gene expression was controlled by other mechanisms such as nucleosome positioning, DNA binding regulatory proteins, and histone modifications. Nonetheless, profiling transcriptional trends by APAs combined with gene expression at single-cell resolution, provides a unique strategy to identify potential prognostic signatures.

## Conclusions

We suggest a computational strategy to profile transcriptional regulation by alternative 3’UTR changes among individual cells, together with gene expression. Our study represents the first analysis of APA variations in combination with gene expression using scRNA-seq data and highlights the utility of APA-associated signatures for improving prognostic performance.

## Supporting information

S1 Fig Different APA patterns between tumor and non-tumor cells defined by Roar. Hierarchical clustering was performed using APA estimates (roar value calculated by Roar) (a) for genes and (b) gene sets on individual cells derived from breast cancer patients. Only 461 cells with at least 983 (10% of total genes) detected genes were selected for further analysis. A total of 3,262 genes and 4,754 gene sets showed the signals of APA on 461 cells. Among theme, 922 genes and 808 gene sets were selected as variable components for hierarchical clustering (see Methods).
(EPS)

S2 Fig Example of read density and corresponding 3’UTR shortening. Sashimi plot produced with Integrative Genomics Viewer (IGV) of two alignments for representative single-cell and Bodymap samples for tumor and normal breast. We selected *SET*, *HSP90AA1*, *YWHAZ*, and *RHOA* for presentation, those genes with the strongest shortening specific to each cell type in breast cancer.
(EPS)

S3 Fig Comparision between APA signals and gene expression in TCGA BRCA. No changes in APA signals along the expression level of target genes in BRCA tissue samples. The APA was predicted by DaPars algorithm from the TCGA BRCA versus matched normal breast tissue samples.
(EPS)

S1 Table Overview of the collected single-cell RNA sequencing dataset. (XLSX)

S2 Table List of 1176 gene sets with significantly altered APA and concurrent expression specific for five cell types. (XLSX)

S3 Table Full list of significantly switched gene sets in each cancer type. (XLSX)

S4 Table Correlation and odds ratio results for 53 cell type-specific gene signatures. (XLSX)
